# Effects of UV Stabilizers on Polypropylene Outdoors

**DOI:** 10.3390/ma13071626

**Published:** 2020-04-01

**Authors:** Witold Brostow, Xinyao Lu, Osman Gencel, Allison T. Osmanson

**Affiliations:** 1 Laboratory of Advanced Polymers and Optimized Materials (LAPOM), Department of Materials Science and Engineering, University of North Texas, 3940 North Elm Street, Denton, TX 76207, USA; allison.osmanson@gmail.com; 2 Department of Civil Engineering, Faculty of Engineering, Bartin University, 74100 Bartin, Turkey; osmangencel@gmail.com

**Keywords:** Polypropylene, polymer thermal stability, UV stabilizer, light stabilizer, Nano-ZnO, coatings

## Abstract

Hindered amine light stabilizers (HALSs) and nano ZnO were used to stabilize polypropylene (PP) film-based formulations that were exposed to ultraviolet (UV) light for different lengths of time, simulating the harsh outdoor weather of Dallas, Texas, USA. UV doses applied in our laboratory are 121 times larger than the UV dose provided by the sunlight in Texas. 15 different compositions were studied. Tensile behavior, UV transmittance, thermal stability (by thermogravimetric analysis) and dynamic friction of the so exposed PP-based films were determined. Scanning electron micrographs of fracture surfaces were obtained. Nano-ZnO-containing stabilizers impart strong UV resistance to our films. The combination of HALSs and nano-ZnO stabilizers makes the PP films harder—which is important for some PP applications, such as toy manufacturing.

## 1. Introduction

Light stabilization of plastics has posed a challenge within the wire and cable industry for years [1]. Polymers in general, and especially polypropylene (PP), are highly sensitive to degradation processes when exposed to oxidant atmospheres and ultraviolet (UV) light [2]. Hence, industrial formulations require the addition of UV stabilizers to preserve the physical, mechanical and thermal properties for long periods of exposure. The aim of this study is the determination: which kind of UV stabilizer improves the UV resistance of PP effectively. 

Hindered amine light stabilizers (HALSs) are one of the most important thermal/light stabilizing agents of polymeric materials. They are widely available, with low toxicity and low cost, and are compatible with a broad range of commercially significant polymeric materials. HALSs have a good photo stabilizing effect on organic materials, which may be 2–4 times higher than the effect of conventional ultraviolet stabilizers [3,4]. One explanation of the mechanism of action of HALSs stabilizers on PP is their inhibition of the degradation of the polymer—which has started the formation of free radicals—rather than absorbing UV radiation. HALSs have low volatility and high extraction resistance [5]. Another advantage is that they provide a significant level of stabilization at relatively low concentrations [6]. HALSs with high molecular weight undergo thermal degradation at elevated temperatures, and are colorless. 

**Figure 1:**
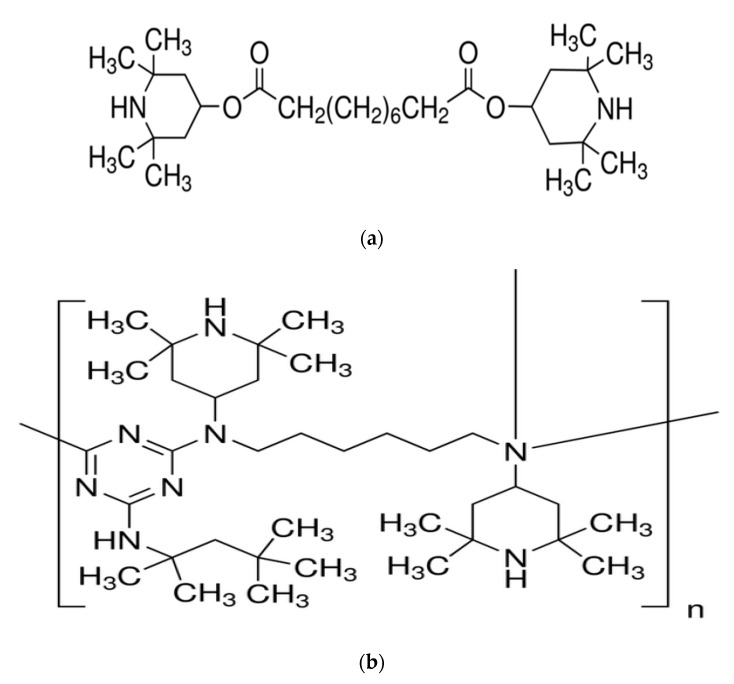
Structure of HALS1 (**a**) and HALS2 (**b**) [7,8].

Figure 1 shows the chemical structures of two kinds of HALSs materials we used.

Chemical scavenging of alkyl and peroxy macroradicals is considered to be the most important process in the mechanism of polymer stabilization by HALS [9]. It is believed that during UV exposure, amine and aminoether derivatives of HALSs yield nitroxides—which act as interceptors of alkyl radicals to generate aminoethers. These aminoethers react with peroxy radicals to recreate nitroxides [10]. 

**Figure 2:**
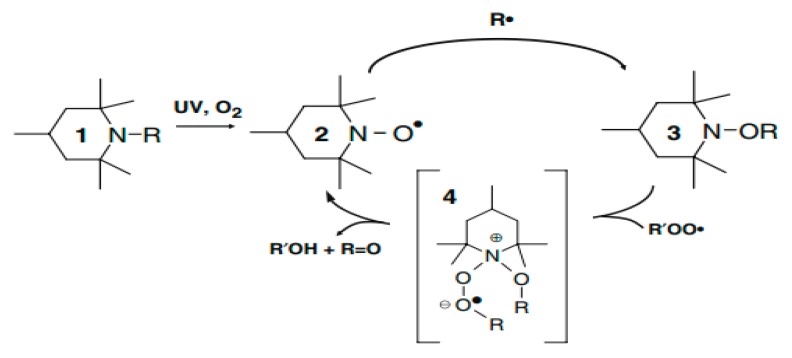
Reaction cycle of HALSs stabilizers.

Figure 2 shows the reaction cycle of HALSs. The inter-conversions of HALSs of various structures operate in a cyclic pathway.

HALS compound (1) is converted into the corresponding nitroxyl radical (2) as the reactive species, which then traps a free radical under the formation of an amino ether function (3). Then (3) interacts with a peroxide radical under the formation of intermediate structures (4), which then decompose into harmless alcohols and ketones while the nitroxyl radical (2) is re-formed. In polymeric media this reaction is probably controlled by molecular diffusion. There are of course various ways to increase UV radiation resistance of polymers [11].

Another ultraviolet stabilizer is nano-ZnO. Nano-ZnO is widely used as an additive in numerous materials and products, including rubbers, plastics, ceramics, glass, cement and lubricants [12]. ZnO is a wide-bandgap semiconductor in the II-VI semiconductor group. The forbidden band gap would increase to 4.5 eV (equal to the most of energy of UV radiation), which causes the absorption ability of nano-ZnO to UV light to increase. The native doping of the semiconductor due to oxygen vacancies or zinc interstitials is n-type [13]. 

Micronized and nanoscale zinc oxide and titanium dioxide provide strong protection against ultraviolet radiation, and are used in suntan lotion, and also in UV-blocking sunglasses for use in space, and for protection when welding—due to research discoveries made by scientists at the Jet Propulsion Laboratory (JPL) in Pasadena, California [14]. The theory behind the use of nano-ZnO as a UV stabilizer involves preventing the formation of cracks in the polymer. When compared with to nano-TiO_2_, nano-ZnO is more effective in diminishing the likelihood that the polymers will begin to yellow [15]. 

## 2. Experimental

### 2.1. Materials 

Polypropylene (EP315J), offered by LyondellBasell, wire and cable grade, has the melt flow rate 2.6 g/10 min, density 0.9 g/cc, tensile strength 3,200 psi, tensile elongation at break 600 %, while its ductile–brittle impact transition temperature is ≈ 30 °C. HALS1 is [bis(2,2,6,6-tetramethyl-4-piperidyl)] sebacate from Sigma-Aldrich, CAS number 52829–07–9. It is a white-colored powder, has the melting temperature between 82 and 85 °C, and the degradation temperature 350 °C. HALS 2 is [poly[[6-[(1,1,3,3-tetramethylbutyl)amino]--*s*-triazine-2,4-diyl]-[(2,2,6,6-tetramethyl-4-piperidyl)-im- ino]-hexamethylene-[(2,2,6,6-tetramethyl-4-piperidyl)imino], from Sigma-Aldrich, CAS number: 70624–18–9. It is a white-colored powder, the melting temperature is 136–140 °C, and the degradation temperature 300 °C. Nano-ZnO is from Sigma-Aldrich, CAS number: 1314–13–2; a white-colored powder, particle size 50 nm, surface area 10.8 m^2^/g, refractive index = 2.0041, degradation temperature 1975 °C. 

### 2.2. Sample Preparation

Table 1 shows compositions of the samples studied. A control sample was prepared without a UV stabilizer.

### 2.3. Film Characterization

The average thickness values of PP films are provided in Table 2.

In order to evaluate the efficiency of the plasticizers in the PP films, UV doses were determined, tensile testing and thermogravimetric analysis (TGA) performed, dynamic friction determined, and scanning electron microscopy (SEM) observations were made. The tests were conducted at 23 °C ± 2 °C, and at 50% ± 5% relative humidity after conditioning the samples in these same conditions for at least 48 h.

### 2.4. UV Dose Calculation 

The UV dose is measured in millijoules per cm^2^ (mJ/cm^2^). The dose is calculated using the following parameters: UV Intensity (I) in miliwatts per cm^2^ (mW/cm^2^); UV Transmittance (UVT) (%) and Exposure time (under UV lamp) (t) (seconds). The relationship between these parameters can be described by the following simplified equation [16]:

UV Dose = (I/UVT) × t(1)

A BlakRay B-100A high intensity UV Lamp with the wavelength of 365 nm was used, with the intensity 16.9 mW/cm^2^. This while the average UV intensity of the sunlight in Texas was determined in 2016, and amounted to 0.14 mW/cm^2^ [17]. This allowed us accelerated laboratory testing, with the UV dose in our laboratory 121 times larger than that created by the sunlight in Texas. 

### 2.5. Tensile Properties

Tensile strength (TS), tensile elongation at break (ε_b_) and Young’s modulus (E) of the films were determined at room temperature using a Mariana Tensile (TestWorks^@^4, USA) according to the American Society for Testing and Materials (ASTM) D882 standard [18]. Films were cut into dog bone-shaped strips 10 × 5 × 1 mm (testing parts), and mounted between the corrugated tensile grips of the instrument. For the film samples, the initial grip spacing and cross-head speed were set at 50 mm and 0.1 cm/s, respectively. The tensile strength was expressed as the maximum force at break divided by the initial cross-sectional area of the film strip.

### 2.6. Thermogravimetric Analysis (TGA) 

TGA is a technique described in [17] in some detail. In our case it is essential for the determination of the effects of stabilizers included in PP films. Measurements were carried out in a Micromeritics TGA (Micromeritics Instruments Corp., Norcross, GA, USA) in N_2_ atmosphere (50 mL/min) at a heating rate of 20 °C/min. The samples were put into platinum pans and scanned from ambient temperature to 600 °C. After the temperature reached 600 °C, we keep the temperature stable for 1 min before cooling down the sample to room temperature.

### 2.7. Dynamic Friction Analysis 

Tribology is a very broad area that includes the studies of friction, lubrication, wear, adhesion, scratch resistance and any interactions of multiple surfaces [17,19,20,21,22]. Dynamic friction is an important indicator, and has been determined by using a tribometer produced by Nanovea Inc. The testing mode we chose is called the “pin-on-disk” mode. As the name implies, a specimen is secured on a spinning disk, and it is contacted with a stationary pin, which is subjected to normal 5.0 N force while the machine is running. An SS302 stainless steel ball with 3.2 mm diameter has been used as a pin. During the testing, the total sliding distance was 75.36 m (6000 revolutions and a track with 2 mm radius) and the spinning speed was 200 revs/min.

### 2.8. Morphology of Film Surface (SEM) 

Several samples exposed to different temperatures were examined under SEM, using the TM3030 Plus Tabletop Microscope from Hitachi High-technologies Corporation 2014 (Chiyoda, Japan). The surface of each of the samples were scanned to determine the influence of the UV light on surface and microstructure of the samples.

## 3. Results and Discussion 

Stabilized PP (with HALSs, nano-ZnO and HALSs + nano-ZnO combined UV stabilizers) films produced by extrusion show white and homogenous surfaces. The chemical modification of the stabilizer does not show a significant influence in film thickness compared to the unstabilized PP resins. After periods of time under exposure of a UV lamp, HALSs stabilized and unstabilized PP components show a little yellowish homogenous surface. However, nano-ZnO stabilized PP components surface does not change.

### 3.1. UV Transmittance

UV transmittance results are shown in Table 3. 

From Table 3, the transmittance of HALSs materials almost does not change with the loading level, while the UV transmittance of nano-ZnO material is much lower than that of HALSs materials. Thus, pure nano-ZnO material has very strong ability to block the UV radiation out of PP samples. With the loading level increasing, the transmittance of nano-ZnO in PP samples becomes lower.

### 3.2. Tensile Properties

Table 4 shows the comparison of tensile properties for our materials.

**Figure 3:**
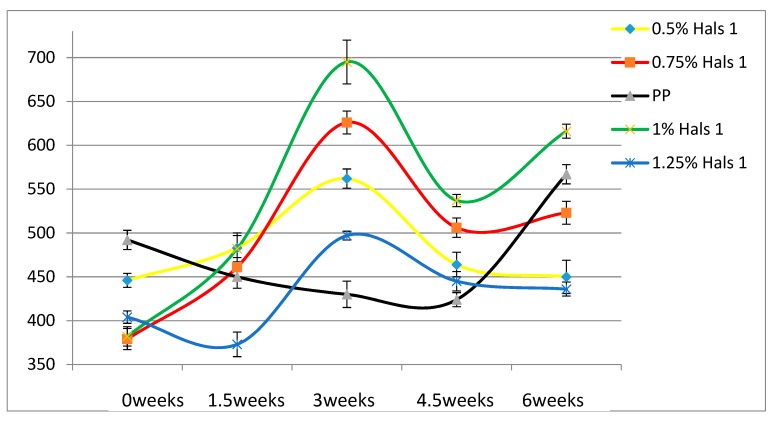
Young’s moduli (MPa) of HALS1 samples as a function of UV exposure time.

**Figure 4:**
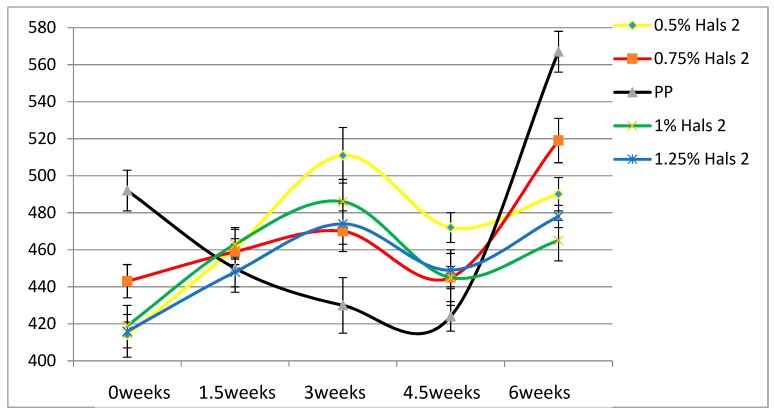
Young’s moduli (MPa) of HALS2 samples as a function of UV exposure time.

**Figure 5:**
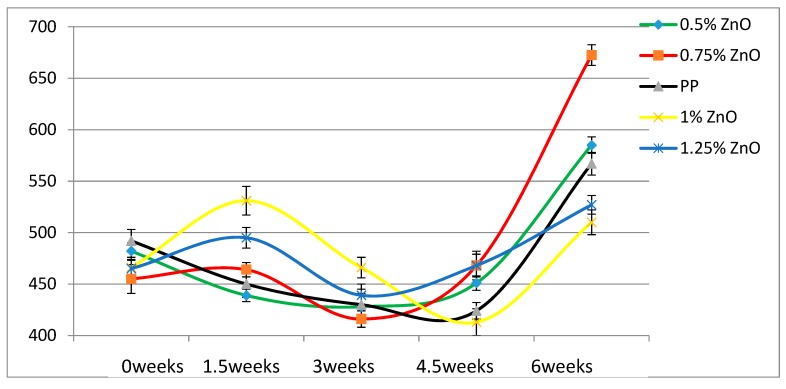
Young’s moduli (MPa) of nano-ZnO samples as a function of UV exposure time.

**Figure 6:**
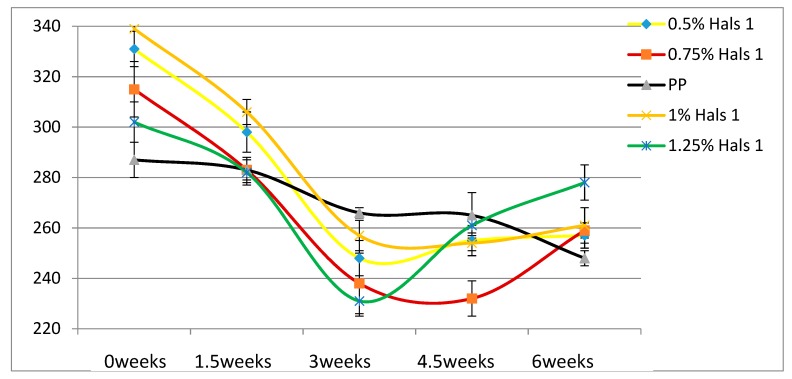
Elongation at break (mm) of HALS1 samples.

**Figure 7:**
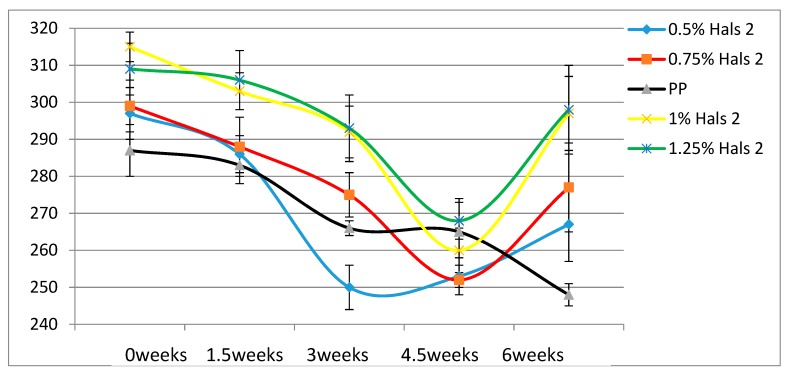
Elongation at break (mm) of HALS2 samples.

According to Table 4, the Young’s modulus of each sample increases as the concentration of UV stabilizers increases. This while there are no significant changes of elongation at break as the concentrations of UV stabilizers increase. As for toughness, the effects of stabilizers are relatively small. These results are also displayed in Figure 2, Figure 3, Figure 4, Figure 5, Figure 6 and Figure 7 as a function of time in weeks. Values for 0 weeks are the same as in Table 4.

Young’s modulus of pure PP film first decreases as UV exposure time increases; after being exposed for 4.5 weeks, pure PP film starts to degrade and to became brittle. 

Elongation at break, ε_b_, shown in pure PP film, in general has been declining as the UV exposure time has been increasing. We recall that that elongation is inversely proportional to the brittleness B [17,23]

B = 1/(ε_b_ × E’) (2)

Here E’ is the storage modulus at 1.0 Hz determined by dynamic mechanical analysis (DMA) [17], both ε_b_ and E’ measured at the temperature of interest. Brittleness is not an inverse of flexibility Y, since the latter is defined [24] as:

Y = V_sp_/ ∑_i_^n^U_bi_ (3)

Here V_sp_ is the specific volume in cm^3^/g at a given temperature while ∑_i_nU_bi_ is the sum of the strengths of bonds in the monomer of a given polymer. 

Both Young’s modulus and the elongation at break trend lines of HALSs-stabilized PP films show a wave-like tendency because of the reaction cycle of HALSs. Slopes of HALS1 and HALS2 samples change before and after 1.5 weeks’ UV exposure; Young’s modulus of HALS1 and HALS2 samples show crests after 3 weeks of UV exposure. This means HALS samples were experiencing steps (2), (3), and (4) in Figure 2 at 1.5, 3 and 4.5 weeks, and would continue to follow the circulation shown in that figure. For pure PP samples, their internal structure would have been destroyed after 4.5 weeks’ UV exposure; however, HALSs and UV stabilizers prevent this from happening. 

**Figure 8:**
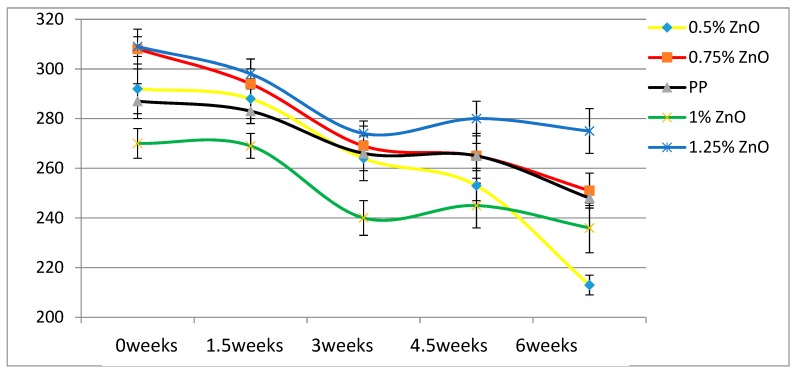
Elongation at break (mm) of ZnO samples.

As the loading level of HALS stabilizers increase, the mechanical properties of PP films become more stable. For nano-ZnO samples we see results in Figure 5 and Figure 8. The tensile modulus rises significantly already at 0.5 wt.% ZnO. Values of the elongation at break for 1.0 % ZnO are lower at all times than those for pure PP; aggregation of the nanopowder particles is a possible explanation. Significant increases of ε_b_ with respect to pure PP are seen at 1.25 % ZnO. At all times the ε_b_ values for that composition are the highest of all. 

Comparing the two HALS-containing PPs at the same stabilizer concentration of 1.25 %, we see that HALS2 provides ε_b_ some 20 mm larger than HALS1. The Young’s modulus values also after 6 weeks and at the same stabilizer concentration are such that HALS2 has the modulus 30 MPa higher than HALS1.

**Figure 9:**
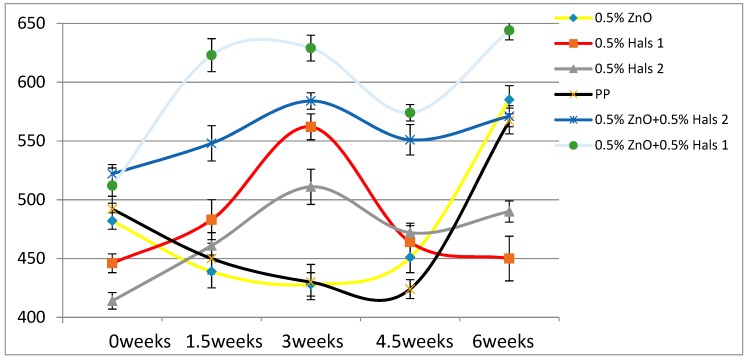
Young’s modulus (MPa) of combined UV stabilizers.

**Figure 10:**
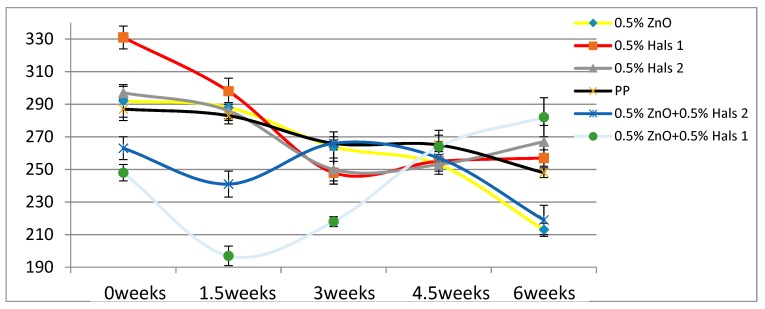
Elongation at break (mm) of combined UV stabilizers.

If HALS and nano-ZnO have synergistic effects and influence each other, there should be a way to prove this relationship. To investigate this, we also made 0.5 wt.% nano-ZnO + 0.5 wt.% HALS1 and 0.5 wt.% nano-ZnO + 0.5 wt.% HALS2 combined stabilizers, and added them to pure PP films. The Young’s modulus and elongation at break of combined UV stabilizers are shown in Figure 9 and Figure 10, respectively. 

Considering the longest exposure time of 6 weeks, we see that both the modulus and the elongation at break are the highest for 0.5 wt.% nano-ZnO + 0.5 wt.% HALS1. As for the composition with 0.5 wt.% nano-ZnO + 0.5wt.% HALS2, it has the modulus comparable to pure PP, but the second lowest elongation at break after 6 weeks. Given Equation (2), this implies the high brittleness B of this composition. We find that there is a synergy effect of nano-ZnO and HALS1, but not in the case of HALS2.

A different property worth consideration is hardness. Compare now the values of the elongation at break after 6 weeks exposure in Figure 6, Figure 7 and Figure 8 and Figure 10, and we see that HALS2 has the highest value of all, ZnO the lowest value. It has been shown that high ε_b_ values for polymers correspond to low Vickers hardness h_V_ values, and vice versa [25]. The relationship is:

hV = 17.61 − 0.0406ε + 2.719 × 10−5ε_b_2 (4)

### 3.3. TGA Results

**Figure 11:**
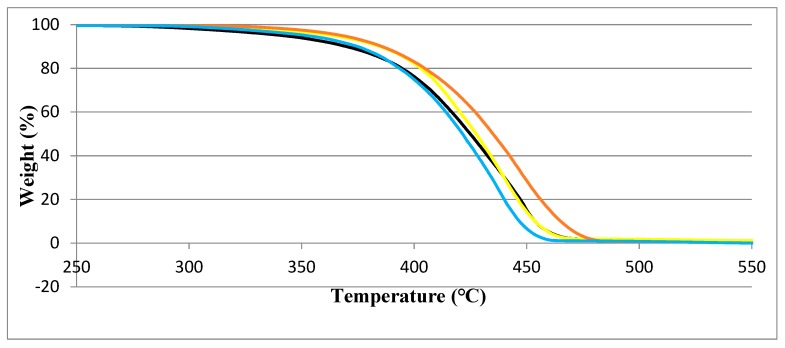
Thermal gravimetric analysis (TGA) results for PP with 1.25 wt.% HALS1 (blue line), PP with 1.25 wt.% HALS2 (red line) and PP with 1.25 wt.% nano-ZnO (brown line).

Shown in Figure 11 are the TGA curves of samples after 6 weeks of UV exposure. We provide curves for the highest concentrations of 1.25 % of all three stabilizers.

We see that different stabilizing additives provide similar effects until 350 °C or so. HALS2 provides a little more stability, since the complete decomposition for the HALS1-containing material occurs above 450 °C, while for the HALS2 system, this takes place some 20 °C higher.

### 3.4. Dynamic Friction Results 

**Figure 12:**
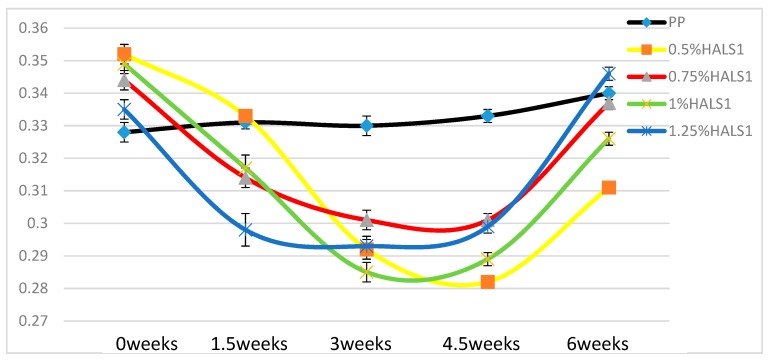
Dynamic friction of pure PP and HALS1 samples.

**Figure 13:**
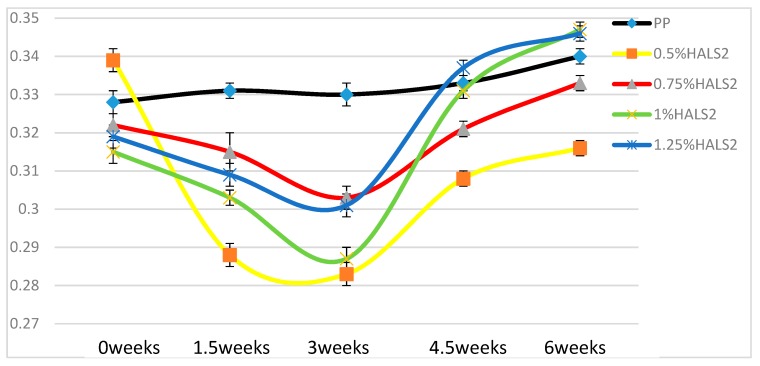
Dynamic friction of pure PP and HALS2 samples.

**Figure 14:**
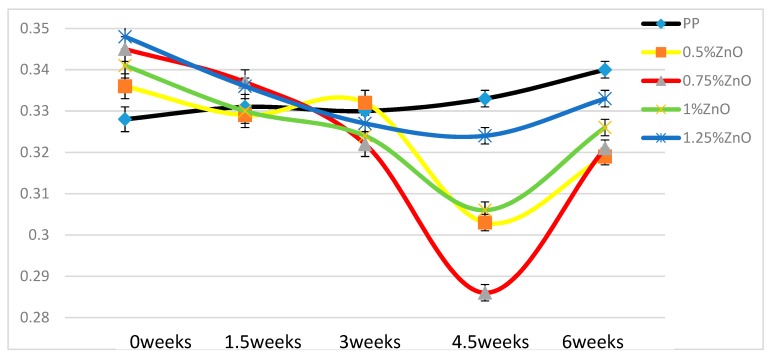
Dynamic friction of pure PP and samples with nano-ZnO samples.

Figure 12, Figure 13 and Figure 14 show the dynamic friction changes for PP films with different kinds of UV stabilizers after different periods of UV light exposure. 

In Figure 12 we can see that surface roughness of pure PP films—as reflected in dynamic friction—continues to increase. This means that under UV light, each of the pure PP film surfaces becomes rougher than before. Adding HALS1 stabilizers increases the surface roughness of PP films without UV exposure, the increase being dependent upon the HALS1 concentration. After periods of UV exposure, the dynamic friction initially decreases. After 4.5 weeks of UV exposure, the friction goes up again, although the values after 6 weeks for samples with 0.5 and 1.0 wt.% HALS1 are clearly lower than the initial ones. However, since the reaction cycle discussed above exists, it is possible that the friction would decrease after another 6 weeks UV exposure. 

In Figure 13 we see similar patterns of behavior with HALS2 as we have seen with HALS1. Curves of dynamic friction for all samples containing HALS2 show minima as a function of times. The friction values are clearly the lowest for 0.5 wt.% HALS2 than for other compositions—except for the time = 0 weeks.

In Figure 14, we can see that the curves for nano-ZnO-containing samples all show minima. All initial values for the nanocomposites are higher than for neat PP. All final friction values after 6 weeks are lower than for neat PP.

### 3.5. SEM Results 

**Figure 15:**
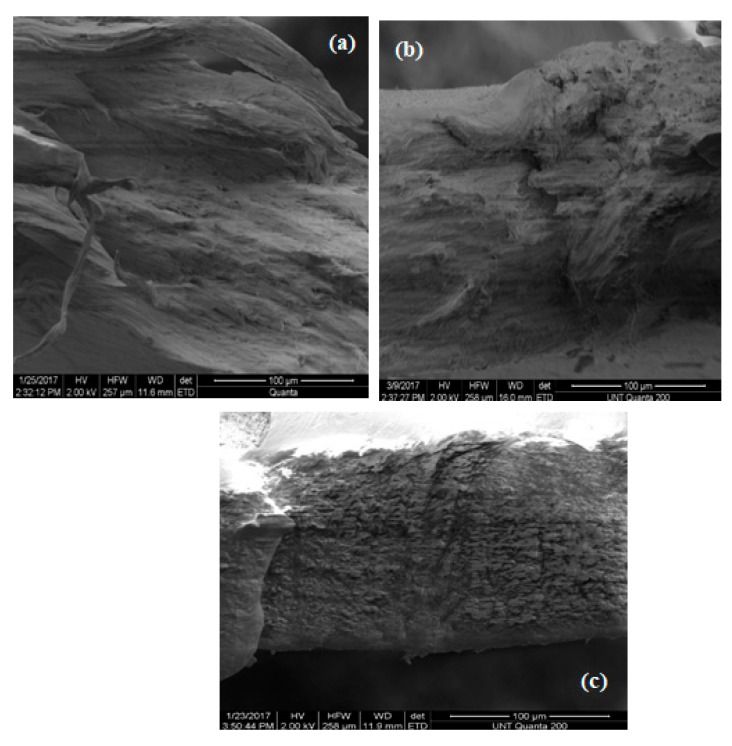
Scanning electron microscopy (SEM) structure of pure PP films.

**Figure 16:**
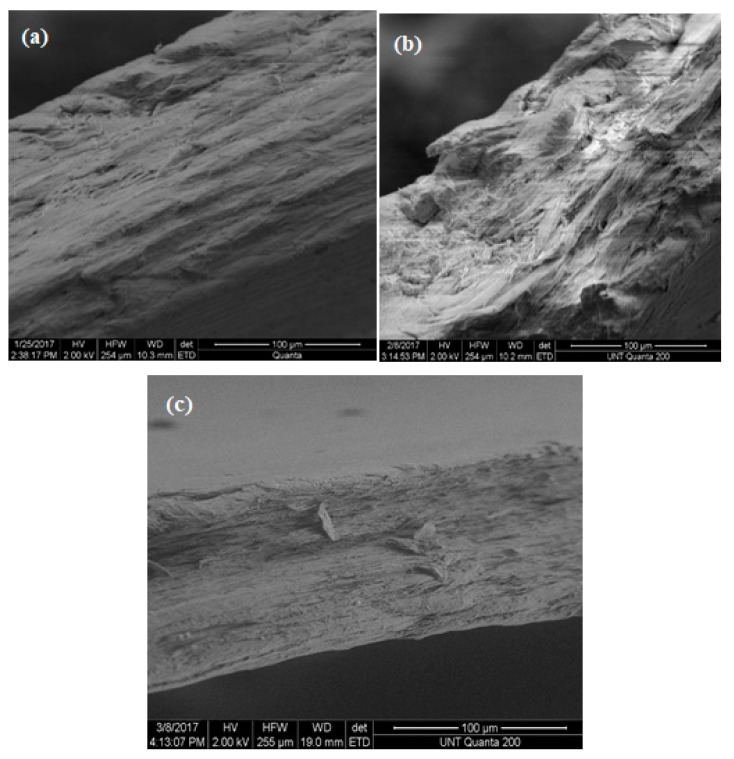
SEM structures of PP films stabilized with 0.5 wt.% HALS2.

**Figure 17:**
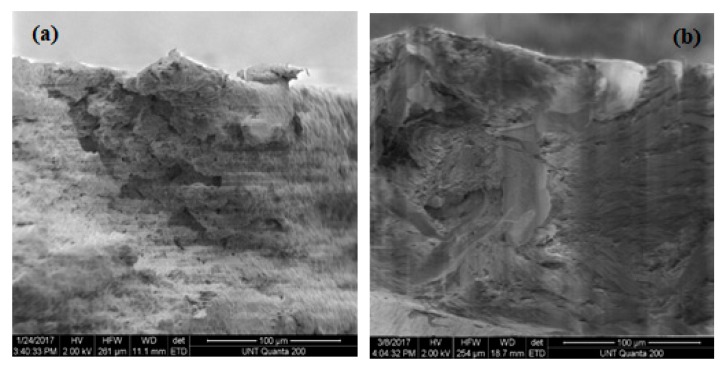

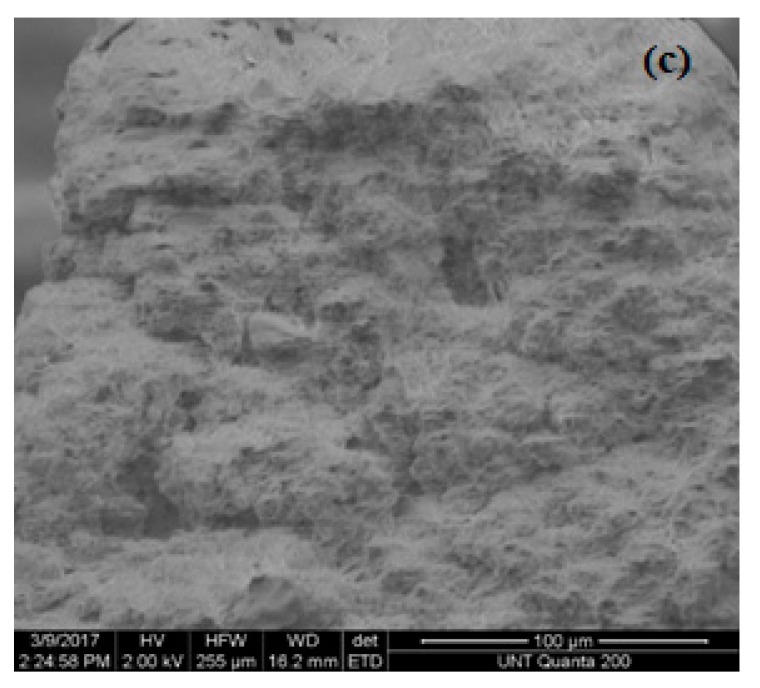
SEM structures of PP films stabilized with 0.5 wt.% nano-ZnO.

Figure 15, Figure 16 and Figure 17 illustrate the SEM structure of fractured surfaces after tensile tests of pure PP, HALS2-stabilized and nano-ZnO-stabilized PP films, respectively. 

In the last three figures, (a) represents films without UV exposure, (b) represents films after 3 weeks’ exposure, and (c) represents films after 6 weeks exposure. For pure PP samples we can see, at first, a compact fiber-like structure—which becomes more granular-like at 6 weeks. Seeing stone-like microstructures of pure PP, we recall decrease with time of values of the tensile elongation at break. Samples with 0.5 wt.% HALS2 show A fiber-like microstructure for all periods of time. Nano-ZnO-containing samples show granular structures at all periods of time. 

## 4. Concluding Remarks 

As discussed by La Mantia and his colleagues [26], “The blend composition can significantly affect the degradative behavior of a polymer blend and can differ from the degradation routes of the pure components since the interactions among different species in the blends during degradation, and among the degradation products, can occur”. We also note that polymers belong to self-organizing materials discussed in detail by Desai and Kapral [27]. 

Nano-ZnO does not affect the mechanical and thermal properties of PP significantly. Only when the loading level is more than 1.25 wt.%, will nano-ZnO show obvious effects on PP. We reiterate that low values of ε_b_ in the ZnO-containing samples correspond to high values of the Vickers hardness.

We find that HALS2 UV-containing stabilizers impart stronger UV resistance to our PP-based films than HALS1 does. Additionally, HALS2 samples can also affect the smoothness of the PP films. Therefore, the HALS2 UV stabilizer is an optimal fit for PP processing in the wire industry. 

To provide a still broader perspective on our results, we note that light resistant polymeric coatings are also used for protection of cultural heritage—as discussed for instance by Andreotti and her colleagues [28]. On the opposite side of the spectrum are situations when we wish the degradation to occur; this is the case of the in vitro biodegradation of iron foams with polymeric coatings studied by Gorejova and her coworkers [29]. 

## Figures and Tables

**Table 1:** Compositions of samples.

	**Samples**						
Components	Control	Ⅰ	Ⅱ	Ⅲ	Ⅳ	Ⅴ	Ⅵ
PP	40 phr	40 phr	40 phr	40 phr	40 phr	40 phr	40 phr
HALS1	N/A	0.50%	N/A	N/A	0.75%	N/A	N/A
HALS2	N/A	N/A	0.50%	N/A	N/A	0.75%	N/A
Nano-ZnO	N/A	N/A	N/A	0.50%	N/A	N/A	0.75%
	**Samples**						
Components	Control	Ⅶ	Ⅷ	Ⅸ	Ⅹ	Ⅺ	Ⅻ
PP	40 phr	40 phr	40 phr	40 phr	40 phr	40 phr	40 phr
HALS1	N/A	1%	N/A	N/A	1.25	N/A	N/A
HALS2	N/A	N/A	1%	N/A	N/A	1.25	N/A
Nano-ZnO	N/A	N/A	N/A	1%	N/A	N/A	1.25

**Table 2:** Thickness of all components.

Components	0.5 wt.% HALS1	0.75 wt.% HALS1	1 wt.% HALS1	1.25 wt. % HALS1
Thickness (mm)	0.5 ± 0.01	0.4 ± 0.03	0.4 ± 0.05	0.5 ± 0.02
Components	0.5wt.%HALS2	0.75 wt.% HALS2	1 wt.%HALS2	1.25 wt.% HALS2
Thickness (mm)	0.5 ± 0.03	0.5 ± 0.04	0.5 ± 0.05	0.5 ± 0.01
Components	0.5 wt.% ZnO	0.75 wt.% ZnO	1 wt.% ZnO	1.25 wt.% ZnO
Thickness (mm)	0.5 ± 0.03	0.5 ± 0.01	0.4 ± 0.03	0.4 ± 0.02

**Table 3:** UV transmittance results.

	UV Transmittance			
	PP	ZnO	HALS1	HALS2
Pure	49%	0.10%	91%	83%
0.50%		28%	49%	49%
0.75%		19%	49%	49%
1%		14%	50%	50%
1.25%		11%	50%	50%

**Table 4:** Comparison of mechanical properties for samples with different concentrations of UV stabilizers.

Concentration	Mechanical Properties	HALS1	HALS2	nano-ZnO
0.5 wt.%	Young's modulus (MPa)	374 ± 31	364 ± 64	429 ± 65
	Elongation at break(mm)	288 ± 47	290 ± 36	279 ± 13
	Tensile toughness (N·m)	9 ± 1	13 ± 2	10 ± 3
0.75 wt.%	Young's modulus (MPa)	379 ± 20	317 ± 57	414 ± 39
	Elongation at break(mm)	295 ± 50	310 ± 64	289 ± 32
	Tensile toughness (N·m)	11 ± 2	14 ± 3	11 ± 2
1 wt.%	Young's modulus (MPa)	382 ± 54	419 ± 23	402 ± 44
	Elongation at break(mm)	321 ± 31	312 ± 28	270 ± 38
	Tensile toughness (N·m)	13 ± 1	12 ± 2	12 ± 2
1.25 wt.%	Young's modulus (MPa)	404 ± 82	444 ± 30	466 ± 70
	Elongation at break(mm)	286 ± 31	276 ± 18	299 ± 50
	Tensile toughness (N·m)	12 ± 2	14 ± 1	12 ± 3
